# Editorial comment: Findings regarding non-sexual penile fracture in a referral emergency hospital

**DOI:** 10.1590/S1677-5538.IBJU.2020.0420.1

**Published:** 2021-02-03

**Authors:** Valter Javaroni

**Affiliations:** 1 Hospital Federal do Andaraí Rio de Janeiro Departamento de Andrologia Rio de Janeiro RJ Brasil Departamento de Andrologia, Hospital Federal do Andaraí Rio de Janeiro, Rio de Janeiro, RJ, Brasil.

## COMMENT

When dealing with a rare condition, it is important to learn from the experience of reference centers. That is the case in this nice paper coming from the biggest public urologic emergency unit in Rio de Janeiro, Brazil (1).

Penile fracture (PF) is one of the less frequent urological traumas and generally has sexual intercourse associated with its mechanism. PF of non-sexual etiology is even rarer, at least in Western countries, and this report adds significant information (2, 3).

First, as a teaching for less experienced emergency physicians, to the possibility of PF in single men in order to properly conduct the situation since the type of therapy is relevant to the outcomes. This is even more significant when considering that mostly of the affected men were in the fourth decade (3).

Secondly, to confirm also in this specific subgroup of PF that its diagnosis is eminently clinical. The typical presentation - the triad of hematoma, detumescence, and snapping sound – is enough to indicate immediate surgical exploration in most cases. Surgical repair of penile fractures was popularized in the 1980s after several studies had demonstrated that long-term complications were reduced from 30% to 4% in surgically treated patients (4, 5). Only in doubtful cases a complementary exam such as an ultrasound or an urethrocystogram (suspicion of urethral injury) shall be performed and justify postpone surgery (6, 7).

But would the time interval between trauma and surgery be a significant variable in relation to outcomes? The answer is surprisingly no, accordingly with authors, that stated in another manuscript: “Even with treatment delay of 21 days, we did not identify a statistical difference between the time of PF repair and complications such as erectile dysfunction or penile curvature rates” (3).

Third, to highlight the value of knowing the level of energy involved in the trauma, since it is related to the surgical findings. In comparison with sexual PF, authors found that in their sample of non-intercourse nor masturbation etiology, bilateral tunica albuginea tears and urethral lesions were less common. And here, another surprising data emerges: complications were similar among those found in their sample of sexual etiology, possible meaning that lower level of energy and less damage did not decrease odds of fibrosis nor erectile dysfunction.

And finally, it is very useful as an alert to properly address the psychological aspect involved in this delicate situation. Most men don't even imagine that their penis can break, especially during sleep. So, it is essential to talk properly before surgery, clarify all doubts and highlight the importance of immediate surgical repair as the best way to minimize sequelae (8).

But as this article and others have shown, even with the ideal treatment, problems can arrive. Late complications of around 10% have been reported in large series of immediately surgically treated penile fractures from reference centers (9). So, this should also be taken into consideration when counseling men with penile fractures regardless of etiology.
